# Effect of metallic materials on magnetic resonance image uniformity: a quantitative experimental study

**DOI:** 10.1007/s11282-024-00778-y

**Published:** 2024-10-15

**Authors:** Hiroaki Shimamoto, Doaa Felemban, Yuka Uchimoto, Nobuhiko Matsuda, Naoko Takagawa, Ami Takeshita, Yuri Iwamoto, Ryoko Okahata, Tomomi Tsujimoto, Sven Kreiborg, Sanjay M. Mallya, Fan-pei Gloria Yang

**Affiliations:** 1 Department of Oral and Maxillofacial Radiology, Osaka University Graduate School of Dentistry, 1-8 Yamadaoka, Suita, Osaka 565-0871 Japan; 2 Division of Oral Radiology, Department of Oral & Maxillofacial Diagnostic Sciences, College of Dentistry, Taibah University, Al Madinah Al Munawwarah, P.O. Box 2898, 43353 Madinah, Saudi Arabia; 3 Department of Pediatric Dentistry and Clinical Genetics, School of Dentistry, Faculty of Health and Medical Sciences, University of Copenhagen, Nørre Allé 20, 2200 Copenhagen, Denmark; 4 3D Craniofacial Image Research Laboratory (School of Dentistry, Department of Applied Mathematics and Computer Science, University of Copenhagen; Centre of Head and Orthopedics, Copenhagen University Hospital Rigshospitalet, Technical University of Denmark), Nørre Allé 20, 2200 Copenhagen, Denmark; 5 Section of Oral and Maxillofacial Radiology, UCLA School of Dentistry, 10833 Le Conte Ave., Los Angeles, CA 90095-1668 USA; 6 Department of Foreign Languages and Literature, National Tsing Hua University, No.101, Section 2, Guangfu Rd., East District Hsinchu, 300013 Taiwan; 7 Center for Cognition and Mind Sciences, National Tsing Hua University, No.101, Section 2, Guangfu Rd., East District Hsinchu, 300013 Taiwan

**Keywords:** MRI, Metallic materials, Image uniformity, Quantitative study

## Abstract

**Objective:**

To assess quantitatively the effect of metallic materials on MR image uniformity using a standardized method.

**Methods:**

Six types of 1 cm cubic metallic materials (i.e., Au, Ag, Al, Au–Ag–Pd alloy, Ti, and Co–Cr alloy) embedded in a glass phantom filled were examined and compared with no metal condition inserted as a reference. The phantom was scanned five times under each condition using a 1.5-T MR superconducting magnet scanner with an 8-channel phased-array brain coil and head and neck coil. For each examination, the phantom was scanned in three planes: axial, coronal, and sagittal using T1-weighted spin echo (SE) and gradient echo (GRE) sequences in accordance with the American Society for Testing and Materials (ASTM) F2119-07 standard. Image uniformity was assessed using the non-uniformity index (NUI), which was developed by the National Electrical Manufacturers Association (NEMA), as an appropriate standardized measure for investigating magnetic field uniformity.

**Results:**

T1-GRE images with Co–Cr typically elicited the lowest uniformity, followed by T1-GRE images with Ti, while all other metallic materials did not affect image uniformity. In particular, T1-GRE images with Co–Cr showed significantly higher NUI values as far as 6.6 cm at maximum equivalent to 11 slices centering around it in comparison with the measurement uncertainty from images without metallic materials.

**Conclusion:**

We found that MR image uniformity was influenced by the scanning sequence and coil type when Co–Cr and Ti were present. It is assumed that the image non-uniformity in Co–Cr and Ti is caused by their high magnetic susceptibility.

## Introduction

Magnetic resonance imaging (MRI) offers several advantages over X-ray-based imaging modalities, such as computed tomography (CT). It provides a broad range of tissue contrasts that cannot be achieved with CT imaging, with no ionizing radiation exposure, and offers the ability to select optional cross-sections [1–3]. However, the spatial resolution is usually lower than that of CT and the scanning times are longer [4].

Currently, MRI is performed using surface coils placed close to the body site being imaged to improve the relatively low spatial resolution [5]. Relative to whole body/volume coils, surface coils provide a much higher signal-to-noise ratio [5]. The size and configuration of the surface coil (i.e., consisting of one or more coils forming a specific configuration) are often optimized for a particular region of interest. Structures of interest in the maxillofacial region are small and pathoses may be subtle, and head and neck (HN) coils are often used to provide better spatial resolution [6]. Many surface coils are phased-array coil type, including the brain (Brain) and HN coils. These coils contain several surface coil elements or channels (each having its receiver), which allow for parallel imaging. This results in a larger area being covered and a reduction in scanning time, while also improving the signal-to-noise ratio [5]. However, the use of surface coils, particularly phased-array coils, may result in a significant loss of overall signal uniformity [5, 7].

The configuration of the surface coil for any imaging examination may significantly impact the diagnostic quality, and potentially, the diagnostic outcome. The strength of the magnetic field induced by the surface coil decreases with distance from the coil. Tissue located closer to the coil provides stronger signals and are emphasized in the image. In contrast, signals received from deeper tissues are weaker. Optimal design and placement of surface coils are important to mitigate this effect. Inappropriate coil selection may cause artefacts that mislead a physician to make inaccurate diagnoses.

It is well-known that magnetic substances, such as primary metals and alloys, alter the local magnetic field strength due to their higher magnetic susceptibility, causing artefacts on MR images. However, to our knowledge, little attention has been focused on an influence of metallic materials on MR image uniformity despite the potential risk of incorrect diagnoses. Surface coils induce local magnetic inhomogeneity, which can exacerbate magnetic susceptibility artefacts. In the head and neck region, this is particularly relevant at the interfaces of two tissues with different magnetic susceptibilities, e.g., air and bone within the paranasal sinuses and the numerous bone–muscle interfaces along the facial skeleton.

One recent study examined the impact of several factors, anticipated to influence image uniformity assessed in accordance with the American Society for Testing and Materials (ASTM) guidelines (i.e., coil type, image correction method, scanning plane and sequence, and metallic material) [8]. However, these authors reported the influence of the metallic material on image uniformity for only one cross-section through the center of the metallic material and did not evaluate slices located away from the metallic material. To avoid misdiagnosis as a result of non-uniform images, it is important to define the three-dimensional extent of signal inhomogeneity caused by metallic materials not only in cross-sections through the metallic materials but also in the vicinity of the metallic materials. To that end, the three main objectives of the present study were as follows: (1) to evaluate the impact of metallic materials on MR image uniformity with two surface coil types, commonly used in head and neck imaging; (2) to evaluate similarities and differences of this impact in three orthogonal tomographic planes; and (3) to evaluate MR image uniformity depending on the two imaging sequences, in the presence of metallic materials. These three objectives are critical irrespective of the presence of metallic materials in the scanned area.

## Materials and methods

### Phantom

The imaging phantom is a specially ordered cubic phantom (dimension: 15 × 15 × 15 cm) made of glass (SiO_2_) and filled with 5% copper sulfate (CuSO_4_) solution, as recommended by ASTM [9], because its T1 and T2 relaxation times and proton density are well established. The volume of the cubical phantom represents the approximate anatomic dimensions of the maxillary and mandibular dentoalveolar regions.

### Metallic material

**Fig. 1:**
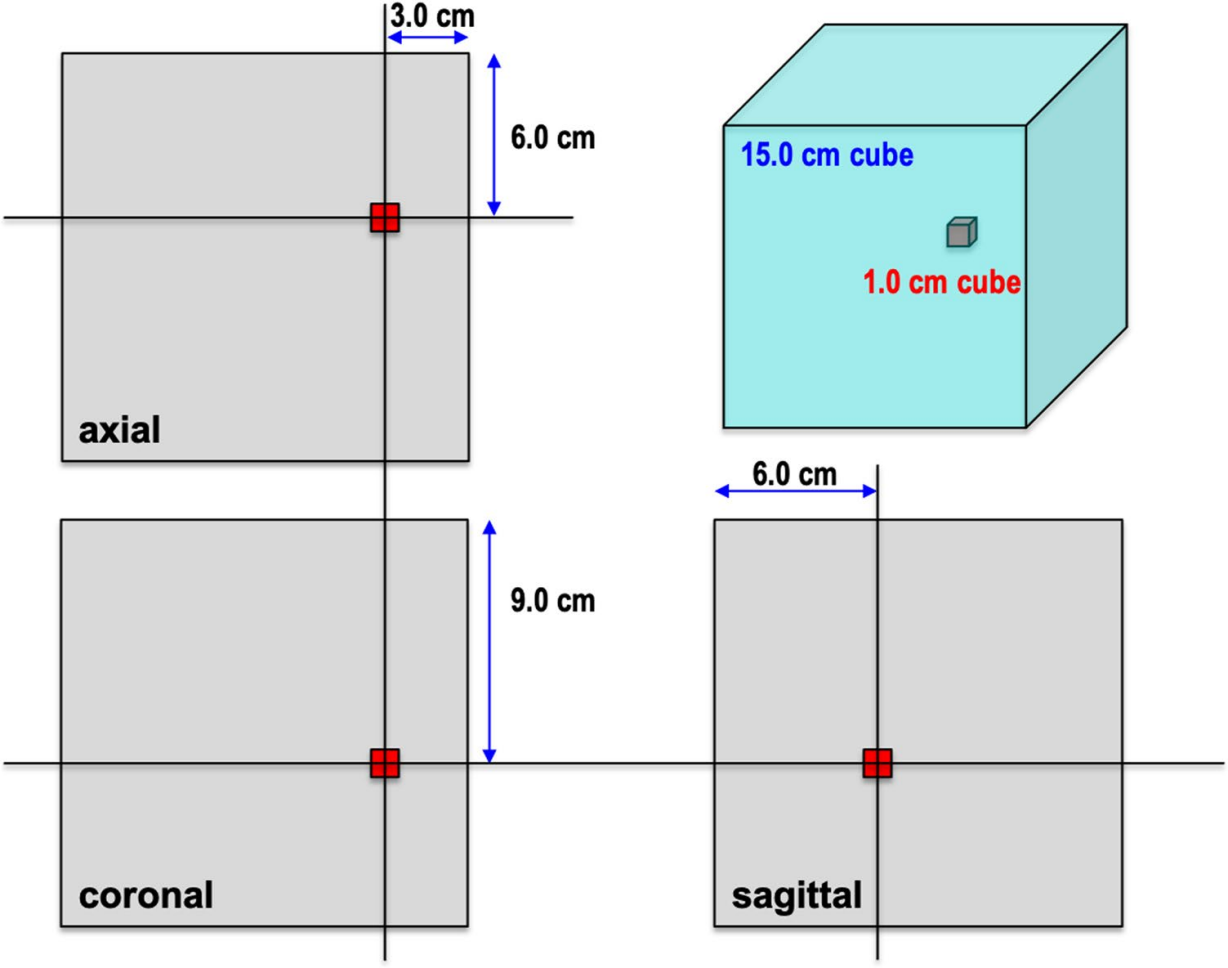
Graphical representation of the position of the metal sample inside the phantom

To assess the impact of metallic materials on MR image uniformity, six commonly utilized metallic dental materials were employed, specifically: Gold (Au), Silver (Ag), Aluminum (Al), Gold-Silver-Palladium (Au–Ag–Pd) alloy, Titanium (Ti), and Cobalt–Chromium (Co–Cr) alloy. Table 1 shows a detailed overview of the characteristics of the sample materials. In accordance with ASTM standards, each sample was represented by a 1 cm^3^ cube suspended by a nylon rod within the cubic phantom individually [9, 10]. All metal cubes were made to specifications by Kojundo Chemical Laboratory Co., Ltd (Saitama, Japan). To simulate a clinical situation, the position of the sample in the phantom (i.e., *x* = 3 cm, *y* = 9 cm, *z* = 6 cm) represents the approximate anatomic location of the left molar area in a typical human subject (Fig. 1).

**Table 1:** Composition of the metallic materials and their magnetic susceptibility

Pure metal	Element symbol	Magnetic susceptibility (× 10^−6^ cm^3^ mol^−1^)
Gold	Au	− 28.0
Silver	Ag	− 19.5
Aluminum	Al	16.5
Titanium	Ti	151.0
Chromium	Cr	167.0
Cobalt	Co	Ferromagnetic
Copper	Cu	− 5.5
Palladium	Pd	540.0
Molybdenum	Mo	72.0
Alloy metal	Composition	
Au–Ag–Pd alloy	Au (12%), Ag (51%), Pd (20%), Cu (15%)	
Co–Cr alloy	Co (63%), Cr (30%), Mo (5%)

### MRI

The assembled phantom was scanned five times by D.F. (four experience years) and average + standard error was reported by H.S. (20 experience years) using the following coils: (1) Brain coil; 8-channel high-resolution coil (GE Healthcare: 2,317,112-2, Milwaukee, WI), and (2) HN coil; 8-channel neurovascular coil (GE Healthcare: 800,121, Milwaukee, WI) on the cradle of a 1.5-T superconducting magnet scanner (GE Signa^®^ HDxt 1.5 T; GE Healthcare, Milwaukee, WI). Imaging parameters were selected in accordance with ASTM-F2119-07 standards [9]. The number of slices was 26, with a thickness of 5 mm each and the interval of 1 mm between slices. For each examination, the phantom was scanned in the axial (perpendicular to the main magnetic field), coronal (parallel to floor), and sagittal planes (perpendicular to axial and coronal planes), using spin echo (SE) and gradient echo (GRE) sequences. The following parameters were employed—field of view: 20 cm by 20 cm, matrix size: 256 by 256 (i.e., pixel size; 0.8 mm), repetition time: 500 ms, echo time: 10 ms, number of excitations: 1, flip angle in GRE: 30° (in accordance with ASTM-F2119-07 standards) [9]. The center of the magnetic field corresponds to images No. 13–14 in the three orthogonal planes. The cross-section through the center of the metallic materials corresponds to images No. 16 image in the axial plane, the No. 11 in the coronal plane, and the No. 6 in the sagittal plane.

### Uniformity evaluation

**Fig. 2:**
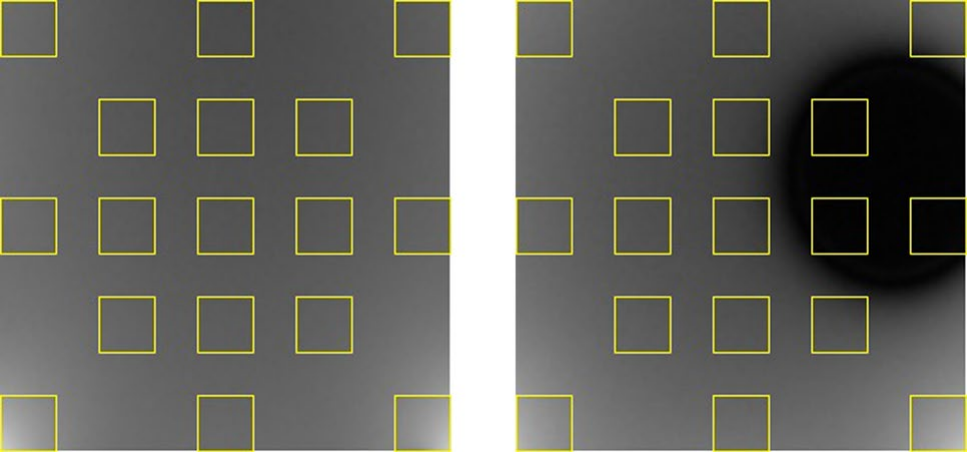
Left: standardized 17-point measurement method based on NEMA when no metallic material is present. Right: standardized 17-point measurement method based on NEMA when Co-Cr was present in GRE sequence using Brain coil. *NEMA* national electrical manufacturers association

All images were imported into the FIJI app (ImageJ, version 2.0.0-rc-69/1.52p) that could adjust the images’ characterization to reach the requirements of image analysis as specified by NEMA (National Electric Manufactures Association) [11]. To assess the uniformity of the images, we quantified the signal intensities (SI) at 17 sample points in accordance with the standardized sample method delineated by NEMA (Fig. 2). This approach enables the calculation of a non-uniformity index (NUI) for each image (see Eq. 1) [11].

1 $$ {\text{NUI}}\, = \,{\text{SImax}}\, - \,{\text{SImin}}/{\text{SImax}}\, + \,{\text{SImin}}\, \times \,{1}00 $$

The equation used in deriving NUI is based on SI, whereby 100% is completely non-uniform and 0% is absolutely uniform [11].

### Statistical analyses

For NUI comparisons between two groups (between SE and GRE sequences and between Brain and HN coils), we used the Mann–Whitney *U* test using SPSS ver. 22 (IBM Corp., Armonk, NY) with the null hypothesis that there were no significant differences between the groups (significance levels set at *p* < 0.01). For NUI comparisons between the three groups (between three scanning planes), we used the Kruskal–Wallis test assuming no significant difference between the groups as the null hypothesis (significance: *p* < 0.01). Furthermore, post hoc pairwise analysis was performed using a Mann–Whitney *U* test with Bonferroni’s correction, with a *p* value of 0.0033 (0.01/3) indicating a significant difference. For the NUI comparisons among seven groups (i.e., six metals and a no metal groups), ± 2 SD in the five times NUIs without the presence of metallic materials was calculated as the measurement uncertainty. NUIs over the measurement uncertainty were defined as significant image non-uniformity produced by the presence of metallic materials.

## Results

Each MR acquisition yielded 26 slices. For each dataset, slice numbers 1–3 and 24–26, located at the phantom edges, were excluded from the analysis to account for minor variations in phantom position within the scan volume.

### ***Results without metallic materials (***Figs. 3, 4, and 5***)***

**Fig. 3:**
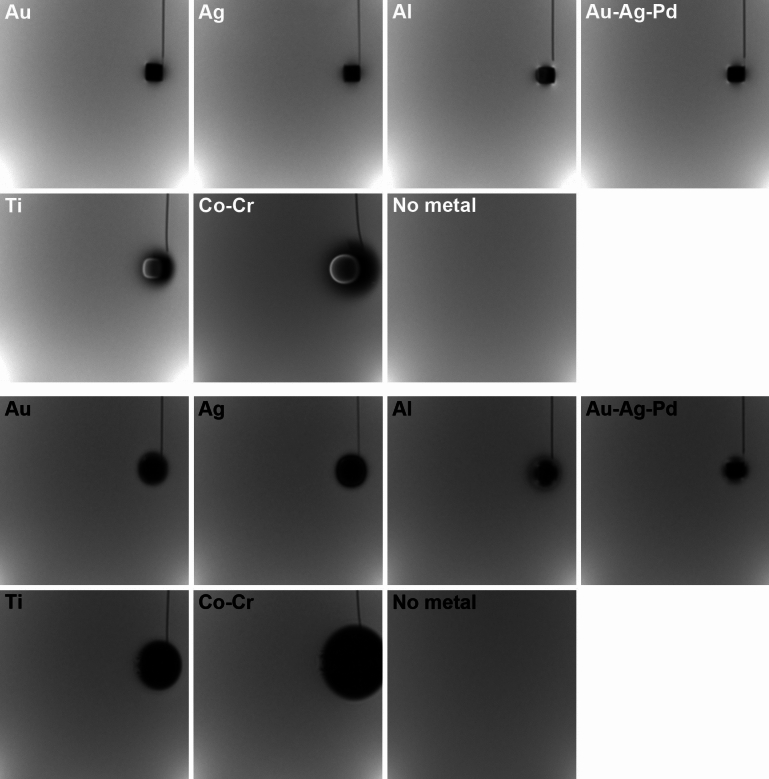
Representative artifacts from metallic materials using Brain coil (axial images). White characters: SE sequence, black characters: GRE sequence

**Fig. 4:**
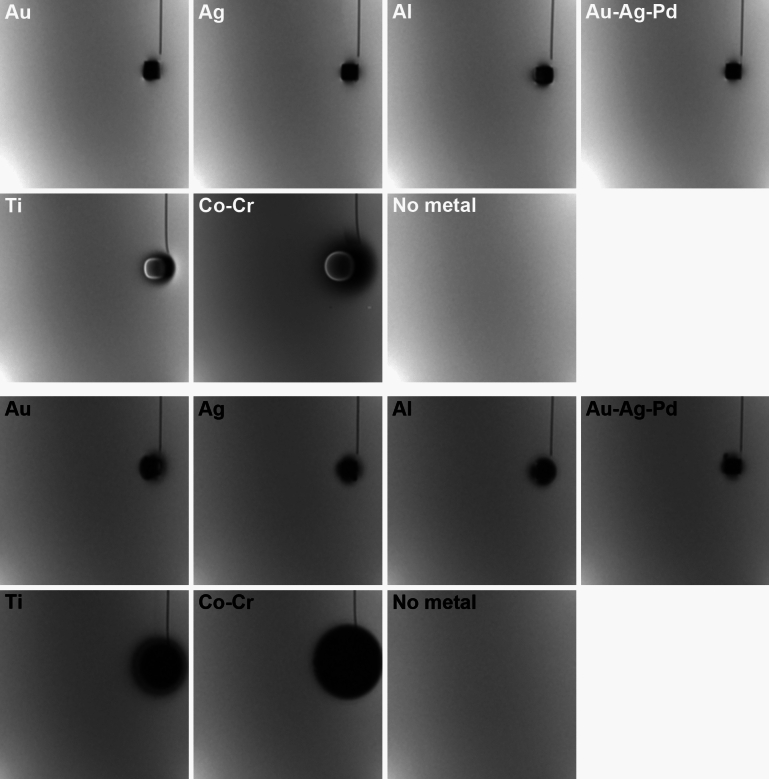
Representative artifacts from metallic materials using HN coil (axial images). White characters: SE sequence, black characters: GRE sequence

**Fig. 5:**
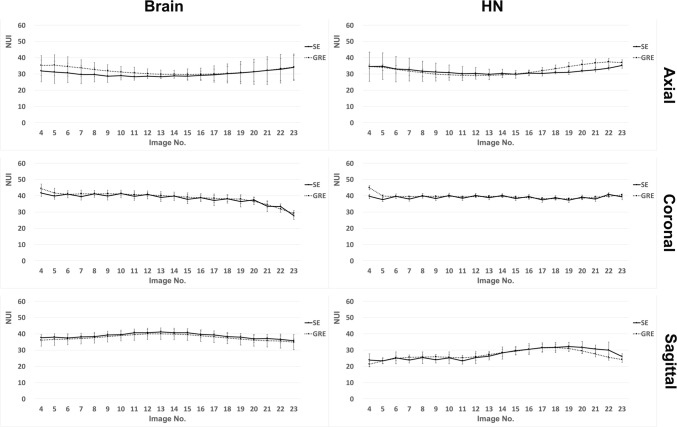
Graph showing the NUI measured at each image slice (4 to 23) in the absence of metallic materials. MR images of the phantom were acquired with the SE and GRE sequences and NUI was assessed on individual slices in accordance with the 17-point measurement method. The coil type (Brain and HN) and scanning planes (axial, coronal, sagittal) are indicated. Lower NUI values indicate the better uniformity. The center of the magnetic field corresponds to No. 13–14 images in the three orthogonal planes. The cross-section through the center of the metallic materials corresponds to the No. 16 image in the axial plane, the No. 11 image in the coronal plane, and the No. 6 image in the sagittal plane. *NUI* non-uniformity index

First, we evaluated MR image signal homogeneity in the absence of metallic materials. The patterns were similar between the two scanning sequences (SE vs. GRE), and the coil type and scanning plane did not impact image uniformity (*p* = 0.056 at minimum). In addition, the patterns were similar between the two coils (Brain vs. HN), and there was no significant difference (*p* = 0.151 at minimum) on each axial image, irrespective of the scanning sequence. Similarly, there was no significant difference (*p* = 0.016 at minimum) between the two coils on each coronal image irrespective of scanning sequence, except for significantly low NUI values (*p* = 0.008) on only a few marginal coronal images (No. 21–23) when the Brain coil was used. However, the HN coil showed significantly low NUI values (*p* = 0.008) on most sagittal images, irrespective of the scanning sequence. The Mann–Whitney *U* test with Bonferroni’s correction indicated that the three scanning planes showed similar uniformity patterns irrespective of scanning sequences or coil type (*p* = 0.008 at minimum).

### ***Results with metallic materials (***Figs. 3, 4, and 6***)***

**Fig. 6:**
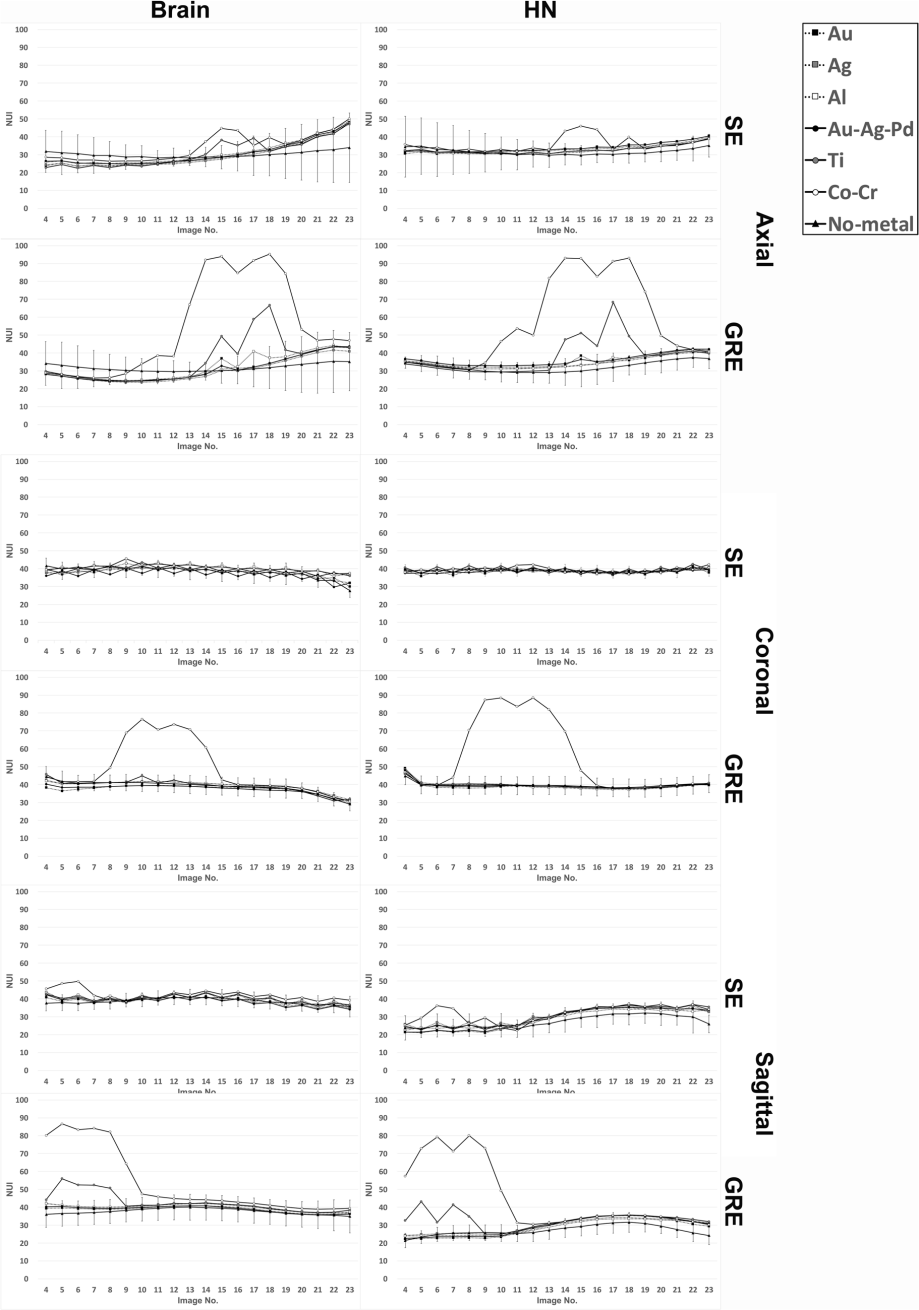
Graph showing the NUI measured at each image slice (4 to 23) in the presence of metallic materials. MR images of the phantom were acquired with the SE and GRE sequences and NUI was assessed on individual slices in accordance with the 17-point measurement method. The coil type (Brain and HN) and scanning planes (axial, coronal, sagittal) are indicated. The slices with the metallic material are described in the legend to Fig. 5. *NUI* non-uniformity index

The presence of metallic materials created artefacts and markedly impacted image uniformity. Compared with GRE images, SE sequence images had lower NUI values adjacent to Co–Cr irrespective of coil type or scanning plane (*p* = 0.008). Similarly, SE generally showed low NUI values adjacent to Ti compared with GRE (*p* = 0.008). The patterns were similar between the two coils (Brain vs. HN), and there was no significant difference (*p* = 0.016 at minimum) on the axial images near Co–Cr and Ti, irrespective of the scanning sequence. However, coronal images made with a Brain coil yielded images with lower NUI near Co–Cr, relative to the use of a HN coil (*p* = 0.008), irrespective of the scanning sequence. On the other hand, sagittal images generated with the HN coil showed lower NUI relative to images mage with the Brain coil (*p* = 0.008), and were observed for both Co–Cr and Ti, irrespective of the scanning sequence.

It was impossible to compare NUI between the three scanning planes because the cross-section through the center of the metallic materials differed among them (image No. 16 in the axial plane, No. 11 in the coronal plane, and No. 6 in the sagittal plane).

Among the six metals used, Co–Cr elicited the highest NUI values, followed by Ti, evident in the GRE sequence irrespective of coil type or scanning plane. In particular, with the GRE sequence, the presence of Co–Cr caused high NUI values at sections as far as 6.6 cm, with values much higher than those in control images without metallic materials. Likewise, imaging with the GRE sequence in the presence of Ti also showed significantly high NUI values as far as 2.4 cm relative to images created without metal within the image volume. The other metallic materials (i.e., Au, Ag, Al, Au–Ag–Pd) did not elicit significantly high NUI values and were within the range of uncertainty measured on images without metallic materials.

## Discussion

This study examined a clinically relevant question—How do metallic dental restorative materials impact MR image uniformity? Practically, the situation represents patients with dental restorations who will undergo MRI of the face, jaws, and orbits. Although the susceptibility artefacts caused within the vicinity of the material are recognized, the full extent of these artefacts and their influence on image homogeneity at more distant sites is less well appreciated.

In this study, we investigated changes to image uniformity with different surface coils (Brain vs. HN), scanning planes (axial vs. coronal vs. sagittal) and with two MR sequences (SE and GRE). The study also examined the impact of these parameters on image uniformity in the presence of commonly used dental metallic materials (Au, Ag, Al, Au–Ag–Pd, Ti, and Co–Cr).

In the absence of metals, the two scanning sequences (SE and GRE) did not affect image uniformity irrespective of the coil type used or the selection of the primary scan plane. Interestingly, the SE sequence elicited high uniformity in the presence of Co–Cr and Ti. Furthermore, we found that the two coils (Brain and HN) did not disproportionately affect image uniformity on axial or coronal images irrespective of scanning sequence, with or without Co–Cr and Ti. The only exception was with the use of a rain coil which yielded high uniformity on the coronal images near Co–Cr. On the other hand, the HN coil elicited high uniformity on sagittal images irrespective of the scanning sequence in the presence of Co–Cr and Ti. The selected scanning plane had no effect on image uniformity irrespective of the scanning sequence or coil type in the no metal condition. Lastly, in the presence of Co–Cr and Ti image,non-uniformity was increased, whereas all the other metallic materials did not affect image uniformity.

### Image uniformity assessment

Several methods have been developed to measure image uniformity. For instance, the American College of Radiology offers an alternative, precise calculation method [12]. In addition to the 17-point calculation method using peak deviation non-uniformity employed in this study, the NEMA method provides approaches to produce greyscale uniformity maps and determine the absolute averaged deviation uniformity. Although each method has its own advantages and drawbacks, we elected to employ the peak deviation non-uniformity methodology utilizing 17 points because the NEMA guidelines make this particularly suitable for evaluating image uniformity in surface coils [11].

### Surface coil selection

Another point of discussion is the selection of the MR surface coils. Our results showed that the HN coil elicited high uniformity on sagittal images irrespective of the scanning sequence in the presence of Co–Cr and Ti or not, although the two coils did not affect image uniformity on axial or coronal images irrespective of the scanning sequence when Co–Cr and Ti were present or not with very few exceptions. It seems suitable to obtain a uniform image for this larger region because the HN coil is intended to cover a comprehensive range from the top of the head to the upper chest area. However, the Brain coil still has relatively high image uniformity.

### Scanning *planes* selection

Although the three scanning planes showed similar uniformity patterns irrespective of scanning sequences or coil type in the absence of metallic materials, it was impossible to compare NUI statistically between the three scanning planes because the cross-section through the center of the metallic materials differed among them. However, in the presence of Co–Cr and Ti, the increase in NUI value was greatest in the axial plane, followed by the sagittal and coronal planes irrespective of coil type or scanning sequence. In this study, the coronal planes were considered to be the most metal-insensitive plane.

### Scanning sequence selection

In this study, two commonly used scanning sequences (SE and GRE) were employed. The primary disadvantage of GRE compared with SE is its susceptibility to the inhomogeneity of the magnetic field, particularly when metallic materials are present and exhibit high magnetic susceptibility (Table 1) [13]. In this study, the two scanning sequences did not affect image uniformity in any combination of coil type or scanning plane, and irrespective of the presence or absence of metallic materials. However, GRE elicited low uniformity in the presence of metallic materials with larger magnetic susceptibilities such as Co–Cr and Ti. This is practically relevant. For clinical diagnostic imaging, the presence of metal and its magnetic susceptibility should be ascertained, and the appropriate sequence can then be selected to minimize undesirable impact on MR image uniformity.

### Metallic materials

In clinical practice, a patient’s body, particularly in the oral and maxillofacial region, may contain metals and/or alloys (e.g., dental fillings, crowns and bridges, orthodontic appliances, dental implants and TMJ replacements). Consequently, it is imperative to assess the impacts of prevalent metallic materials on image uniformity. The present study investigated the use of noble metals, including Au and Ag, as well as noble metal alloys, such as Au–Ag–Pd. Base metals such as Ti (and occasionally Al), which are commonly used in medical and dental procedures (e.g., pins, screws and implants, etc.), were also investigated [14]. Finally, Co–Cr alloy, which are commonly used in medical and dental prostheses (e.g., artificial joints and dental implants), was also studied [15]. When any metallic material is set in a strong magnetic field, the material will be magnetized. The precise degree of the magnetization is referred to as magnetic susceptibility (Table 1). Previous studies have pointed out that metallic materials can cause large metallic artefacts on MRI images; however, whether they would also impair overall image uniformity was not understood [16]. However, Co–Cr elicited the lowest uniformity, followed by Ti with a larger magnetic susceptibility but not for any other metallic materials. The findings indicate that local non-uniformity originating from metallic materials other than Co–Cr and Ti did not significantly affect the overall uniformity in our phantom study. However, our results strongly indicate that the GRE sequence should be avoided in the presence of Co–Cr and Ti.

### Study limitations

In this study, a 15 cm^3^ cubic phantom made of glass (SiO_2_) filled with 5% CuSO_4_ solution was used, in accordance with ASTM recommendations (e.g., well-known T1/T2 relaxation times for CuSO_4_, no artefacts or distortions in glass, etc.) [9]. Although the phantom size was a little smaller than a human head, the size and location of the metallic material (1 cm^3^ in size) were comparable to commonly encountered clinical situations (e.g., a tooth crown in the molar area). However, the phantom-based approach does not account for patient tissue-induced inhomogeneity. Second, patient-specific situations are more complex, for example, patients usually have more dental fillings, crowns, and bridges of various types which may result in different patterns of artefacts and non-uniformity compared with those in this study. Nevertheless, the current results are informative and provide a baseline to examine this issue in general. Perhaps patient scenarios likely to be affected include those with multiple contiguous implants and restorations, full arch prosthesis, etc. Future studies with a larger assortment of metallic materials are needed to better reflect the numerous relevant clinical situations.

### Implications for the dentistry field

The results of this study indicate that non-uniformity was exceptionally high in areas close to metallic materials with high magnetic susceptibility (e.g., Co–Cr and Ti, etc.). This non-uniformity may have little impact on evaluating the presence or gross extensions of a tumor in the oral and maxillofacial region. However, when assessing changes in inflammatory diseases, such as osteomyelitis, mucositis, etc., SI plays an important role as a diagnostic indicator. The use of SE sequence and HN coil selection are recommended when Co–Cr or Ti is in the vicinity of the lesion. In addition, coronal or sagittal imaging may be recommended under these conditions but imaging in the axial plane will likely be non-diagnostic. With the GRE sequence, Co–Cr caused high NUI values as far as 6.6 cm from the center of the material. However, the impact of this inhomogeneity on clinical diagnosis remains to be determined.

## Conclusion

We found that MR image uniformity was adversely influenced by the scanning sequence and coil types when Co–Cr and Ti were present, likely caused by their high magnetic susceptibility.
